# Multicenter analysis of the efficacy of early cholecystectomy and preoperative cholecystostomy for severe acute cholecystitis: a retrospective study of data from the multi-institutional database of the Hiroshima Surgical Study Group of Clinical Oncology

**DOI:** 10.1186/s12876-024-03420-7

**Published:** 2024-10-01

**Authors:** Tomoyuki Abe, Tsuyoshi Kobayashi, Shintaro Kuroda, Michinori Hamaoka, Hiroaki Mashima, Takashi Onoe, Naruhiko Honmyo, Koichi Oishi, Hideki Ohdan

**Affiliations:** 1 Department of Gastroenterological Surgery, National Hospital Organization Higashihiroshima Medical Center, 513, Jike, Saijo-cho, Higashihiroshima, 739-0041 Hiroshima Japan; 2 Department of Gastroenterological and Transplant Surgery, Graduate School of Biomedical and Health Science, Hiroshima University, Hiroshima, Japan; 3 Department of Surgery, Hiroshima Prefectural Hospital, Hiroshima, Japan; 4 Department of Surgery, Onomichi General Hospital, Onomichi, Japan; 5 Department of Surgery, Kure Medical Center and Chugoku Cancer Center, Kure, Japan; 6 Department of Surgery, Hiroshima City Asa Citizens Hospital, Hiroshima, Japan; 7 Department of Surgery, Chugoku Rosai Hospital, Kure, Japan

**Keywords:** Early cholecystectomy, Severe acute cholecystitis, Tokyo guidelines 2018

## Abstract

**Background:**

Severe acute cholecystitis (AC) is a challenging disease because it comprises coexisting systemic infections that lead to vital organ dysfunction. This study evaluated the optimal surgical timing and efficacy of preoperative percutaneous cholecystostomy (PC) for patients with severe AC.

**Methods:**

Data of 142 patients who underwent cholecystectomy for severe AC between 2011 and 2021 were retrospectively collected from the multi-institutional database of the Hiroshima Surgical Study Group of Clinical Oncology. Patients were divided into the early cholecystectomy (EC) group (within 72 h of symptom onset) and delayed cholecystectomy (DC) group. They were also subdivided into the upfront cholecystectomy group and preoperative PC before cholecystectomy group. The diagnosis and severity of AC were graded according to the Tokyo Guidelines 2018. Clinicopathological variables and outcomes were compared.

**Results:**

No significant differences in age, body mass index, American Society of Anesthesiologists (ASA) classification, and Charlson comorbidity index between the EC and DC groups were observed. Preoperative drainage was more commonly performed for the DC group than for the EC group. Local severe AC features were more commonly detected in the DC group than in the EC group. The postoperative outcomes of the EC and DC groups were comparable. Compared to the PC before cholecystectomy group, the upfront cholecystectomy group included more patients with ASA physical status ≥ 3 and more patients who used oral warfarin. Warfarin usage and cardiovascular dysfunction rates of the PC after cholecystectomy group were higher than those of the upfront cholecystectomy group. PC was associated with significantly less intraoperative bleeding and shorter hospital stays.

**Conclusions:**

Patients who can tolerate general anesthesia are good candidates for EC. Patients who use warfarin and those with cardiovascular dysfunction are considered to be at high risk for postoperative complications; therefore, to prevent AC recurrence during the waiting period, PC before cholecystectomy during the same admission is more appropriate than upfront cholecystectomy for these patients.

## Introduction

Acute cholecystitis (AC) is a common disease that requires surgical intervention [1] in accordance with the criteria [2, 3] established by the Tokyo Guidelines 2013. Previous studies and the Tokyo Guidelines 2013 suggested that the optimal timing for surgery is 72 h after symptom onset [4, 5]. AC severity is graded as mild (grade I), moderate (grade II), or severe (grade III), and these grades are associated with postoperative outcomes and the rates of conversion from laparoscopic cholecystectomy (LC) to open cholecystectomy (OC) [6, 7]. The revised Tokyo Guidelines 2018 (TG18) proposed treatment strategies for AC according to its severity [8]. Grade III AC is a systemic infectious condition with local inflammation that is associated with a significantly higher mortality rate than that of grade I and grade II AC [9]. Because organ dysfunction is caused by systemic infections associated with grade III AC, the TG18 recommend initial treatment comprising antibiotics and multidisciplinary methods.

According to the TG18 flowchart for grade III AC, early LC is only recommended when it can be performed at an advanced laparoscopic surgery center, when there is a possibility of organ system failure, and when the patient does not have risk factors that preclude such treatment. LC has several advantages over OC for AC; for example, LC reduces intraoperative bleeding, shortens the postoperative hospital stay, and reduces postoperative complications [10, 11]. Early LC is recommended by the TG18 for grade I and grade II AC; however, the optimal surgical timing for grade III AC is unclear. Early LC is strongly associated with shorter hospital stays and fewer postoperative complications [12]. According to the TG18 treatment strategy for grade III AC, preoperative percutaneous cholecystostomy (PC) is a possible approach for patients with a severe general condition as determined by the ASA classification and Charlson comorbidity index [6]. Because grade III AC presents with systemic infection and local inflammation, a poor general condition complicates the decision of whether to perform upfront surgery or PC before cholecystectomy. Thus, the efficacy of preoperative PC for grade III AC is controversial. During this study, we aimed to determine the optimal surgical intervention and efficacy of preoperative PC for grade III AC.

## Materials and methods

### Patient population and selection

A total of 142 patients diagnosed with AC after cholecystectomy according to the TG18 between May 2011 and August 2021 were enrolled in this study. The measured variables were sex, age, body mass index, American Society of Anesthesiologists (ASA) physical status, and Charlson comorbidity index. Laboratory data and computed tomography (CT) findings were collected preoperatively. The CT findings of the gallbladder wall thickness, abdominal abscess, inflammation around the gallbladder, enhancement of the gallbladder bed, duodenal edema, transverse colon edema, empyema of the gallbladder, ascites, and pleural effusion were collected to analyze nine parameters of AC according to previously reported measurements [13]. A preoperative biliary tract evaluation is a standard procedure that is performed to prevent bile duct injury. Magnetic resonance cholangiopancreatography (MRCP) is the preferred imaging modality for most cases; however, when cholangitis is suspected, preoperative endoscopic retrograde cholangiopancreatography (ERCP) is conducted. When MRCP is contraindicated because of allergies or the presence of metallic implants, CT with intravenous contrast is recommended as an alternative. This study was approved by the local review board, and written informed consent was obtained from all patients.

### Surgical and PC procedures

The consultant surgeon determined whether to perform LC, bailout surgery (BOS), or OC. These operative methods have been described previously [12]. LC was performed using the standard four-port or three-port two-handed technique in the American position. Dissection of Calot’s triangle and the hepatocystic triangle of the gallbladder from the liver bed was performed using monopolar electrocautery. The critical view of safety (CVS) technique, which is valuable to the safe performance of total cholecystectomy, was performed to expose the gallbladder. When CVS was not feasible because of severe inflammation or prior surgical adhesions, BOS was selected. BOS comprised open conversion, subtotal cholecystectomy, and fenestration. PC was performed under local anesthesia using transabdominal ultrasonography. The main reasons for choosing PC were as follows: eight cases were observed in patients who used preoperative direct oral anticoagulants or warfarin; 18 cases were classified as TG18 grade III with associated thrombocytopenia, shock, or prolonged PT; and 14 cases were related to hemodynamic instability or shock. All PC procedures were performed via the transhepatic approach. Subsequently, cholangiography was performed to confirm patency from the gallbladder to the common bile duct a few days later. PC placement was performed based on the discretion of the attending surgeon. A drainage tube was inserted in the gallbladder (8-Fr pigtail) and kept open until the completion of surgery.

### Morbidity and complications

Complications were defined according to the classification described by Clavien et al. [14]. During this study, postoperative complications were defined as those classified as grade IIIa or higher. Hospital death was defined as death that occurred within 90 days postoperatively.

### Statistical analysis

Continuous variables are presented as medians and ranges, and categorical variables are presented as numbers and percentages. Continuous variables were compared using the Mann-Whitney U test, and categorical variables were compared using Fisher’s exact test. To confirm the normality of the data, the Kolmogorov-Smirnov test was performed. Data were analyzed using a one-way analysis of variance. *P* < 0.05 was considered statistically significant. The cutoff values for albumin and C-reactive protein were estimated according to a receiver-operating characteristic curve analysis. Statistical analyses were performed using IBM Statistical Package for Social Sciences for Windows version 22.0 (IBM Corp., Armonk, NY, USA).

## Results

A total of 142 patients enrolled in this study underwent emergency cholecystectomy for grade III AC between April 2011 and December 2021. Of these patients, 91 (64%) were male and 41 (36%) were female. Eighty-five (60%) of these patients were older than 80 years of age at the time of surgery. Grade III dysfunctions were defined as cardiovascular (*n* = 30), neurological (*n* = 20), respiratory (*n* = 14), renal (*n* = 45), hepatic (*n* = 51), and hematological (*n* = 51).

### Comparison of perioperative characteristics of patients with grade III AC in the early cholecystectomy and delayed cholecystectomy groups

Table 1 presents the characteristics of patients with grade III AC after early cholecystectomy (EC) (*n* = 81) and delayed cholecystectomy (DC) (*n* = 61). Significant differences in sex (*p* = 0.02), albumin level (*p* = 0.02), and direct oral anticoagulant usage (*p* = 0.03) were observed. Grade III severity was compared between the EC and DC groups. Preoperative CT findings such as abdominal abscess (*p* = 0.03), inflammation around the gallbladder (*p* = 0.04), ascites (*p* < 0.01), and pleural effusion (*p* < 0.01) were detected more frequently in the DC group than in the EC group. No significant differences in intraoperative bleeding, operative time, or the need for BOS were observed between these groups (Table 2). Postoperative complications with Clavien-Dindo classification III-IV occurred in 12 (15%) patients in the EC group and 8 (13%) patients in the DC group. Three patients in the EC group and three patients in the DC group experienced 90-day mortality. The causes of death were postoperative pneumonia (two cases), interstitial pneumonia exacerbation (one case), hepatorenal syndrome progression (one case), and preoperative septic shock (two cases).

**Table 1:** Characteristics of patients with severe acute cholecystitis after early and delayed cholecystectomy

	Early cholecystectomy (within 72 h) (*n* = 81)	Delayed cholecystectomy (after 72 h) (*n* = 61)	*P*-value
Sex, male/female	45 (56%)/26 (44%)	46 (75%)/15 (25%)	**0.02**
Age older than 80 years	50 (62%)	35 (57%)	0.60
BMI (kg/m^2^)	24 (13–43)	23 (15–36)	0.59
ASA-PS ≥ 3	32 (40%)	24 (39%)	0.98
CCI	2 (0–8)	5 (0–8)	0.31
History of biliary infection	5 (6%)	6 (10%)	0.42
WBC (×10^3^/L)	15,013 (2330-51,900)	13,066 (1490-28,200)	0.39
Hb (g/dL)	12.1 (7.1–16.6)	12.6 (8.6–17.3)	0.32
Alb (g/dL)	3.1 (1.2–4.4)	2.8 (1.5–4.2)	**0.02**
CRP (mg/dL)	20.0 (0.1–45.5)	19.9 (1.4–39)	0.95
Wf usage	10 (23%)	5 (8%)	0.43
DOAC usage	11 (14%)	17 (28%)	**0.03**
Cardiovascular dysfunction	16 (20%)	14 (23%)	0.64
Neurological dysfunction	8 (10%)	12 (20%)	0.10
Respiratory dysfunction	10 (12%)	4 (7%)	0.25
Renal dysfunction	28 (35%)	17 (28%)	0.40
Hepatic dysfunction	30 (37%)	21 (34%)	0.75
Hematological dysfunction	29 (36%)	22 (36%)	0.97
*Preoperative CT findings*			
GB wall thickness	65 (80%)	55 (99%)	0.11
Abdominal abscess	7 (9%)	13 (21%)	**0.03**
Inflammation around the GB	53 (65%)	50 (82%)	**0.04**
Enhancement of the GB bed	35 (43%)	20 (33%)	0.24
Duodenal edema	23 (28%)	23 (38%)	0.24
Transverse colon edema	9 (11%)	12 (20%)	0.16
Empyema of the GB	5 (6%)	6 (10%)	0.39
Ascites	19 (23%)	27 (44%)	**< 0.01**
Pleural effusion	17 (21%)	26 (43%)	**< 0.01**

Abbreviations: Alb, albumin; ASA-PS, American Society of Anesthesiology physical status; BMI, body mass index; CCI, Charlson comorbidity index; CRP, C-reactive protein; CT, computed tomography; DOAC, direct oral anticoagulant; GB, gallbladder; Hb, hemoglobin; WBC, white blood cells; Wf, warfarin

**Table 2:** Perioperative outcomes of patients with severe acute cholecystitis after early and delayed cholecystectomy

	Early cholecystectomy (within 72 h) (*n* = 81)	Delayed cholecystectomy (after 72 h) (*n* = 61)	*P*-value
LC/OC/CC	39/36/6	28/24/9	0.36
Intraoperative bleeding (g)	231 (0-3100)	225 (0-2200)	0.64
Operative time (min)	134 (62–314)	134 (68–263)	0.68
Hospital stay (days)	20 (4–75)	18 (4–89)	0.07
Blood transfusions	12 (15%)	7 (11%)	0.34
Bailout surgery	28 (35%)	24 (39%)	0.56
Clavien-Dindo classification III-IV	12 (15%)	8 (13%)	0.77
BDI	0	1 (%)	0.25
90-day mortality	3 (4%)	3 (5%)	0.72
Gangrenous cholecystitis	48 (59%)	39 (64%)	0.57

Abbreviations: BDI, bile duct injury; CC, conversion cholecystectomy; LC, laparoscopic cholecystectomy; OC, open cholecystectomy

### Comparison of perioperative characteristics of patients with grade III AC in the upfront surgery and PC before cholecystectomy groups

**Fig. 1:**
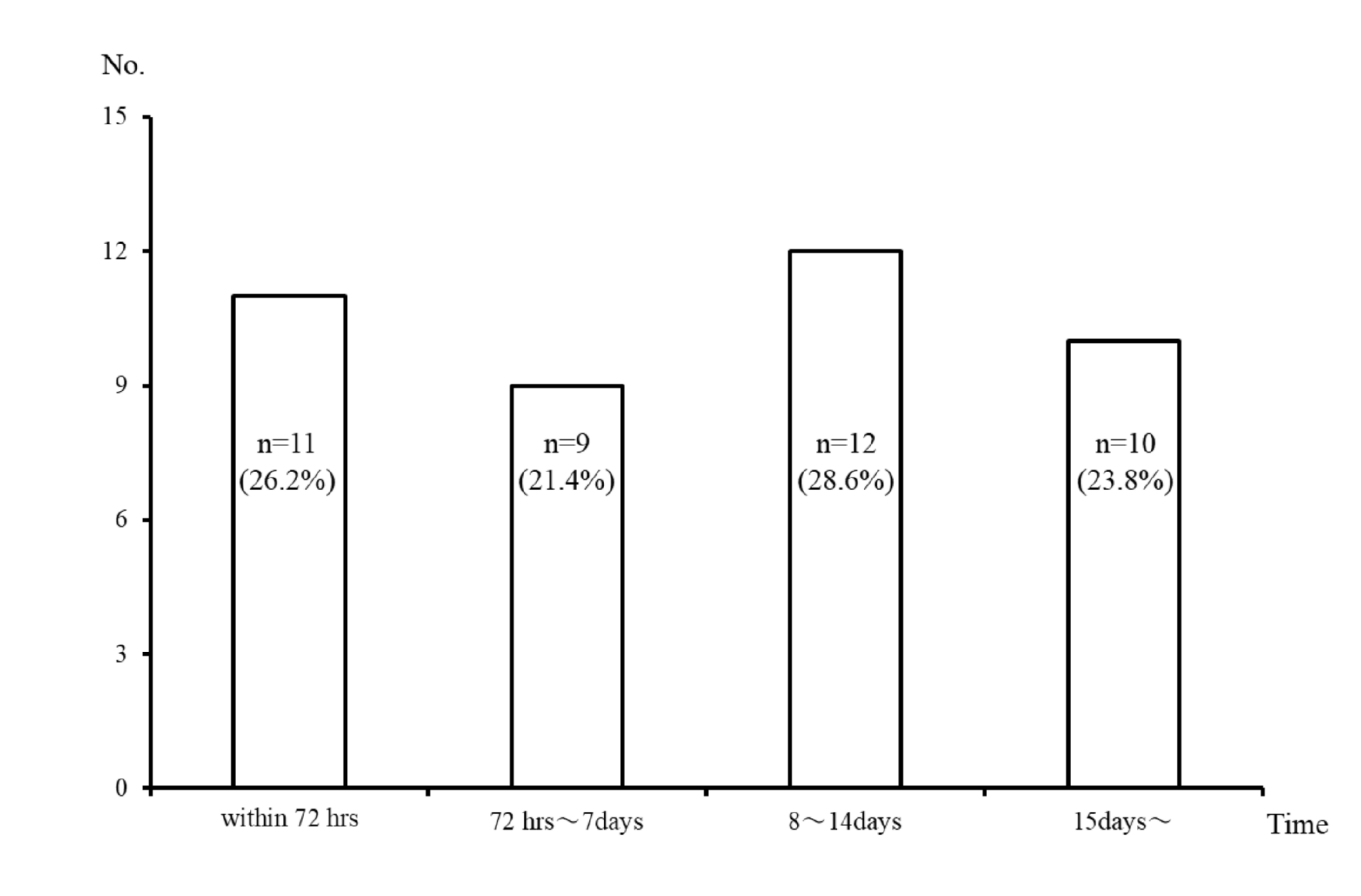
Time from preoperative gallbladder drainage to cholecystectomy

Compared to the EC group, the PC before cholecystectomy group included significantly fewer patients with ASA physical status ≥ 3 (*p* = 0.04) (Table 3). Eight (19%) patients in the PC before cholecystectomy group and seven (7%) patients in the upfront surgery group used warfarin (*p* = 0.03). Among the various types of grade III AC dysfunction, cardiovascular dysfunction (*p* = 0.02) occurred more frequently in the PC before cholecystectomy group than in the upfront surgery group. The preoperative CT findings and surgical types were similar in these two groups. LC was performed for 44 (44%) patients in the upfront surgery group and 23 (55%) patients in the PC before cholecystectomy group (Table 4). BOS was performed for 44 (44%) patients in the upfront surgery group and 17 (40%) patients in the PC before cholecystectomy group. The intraoperative bleeding volume of the PC before cholecystectomy group was lower than that of the upfront surgery group (*p* = 0.04). Although the operative times of these two groups were equal, the length of the hospital stay of the PC group was significantly shorter than that of the upfront surgery group (*p* < 0.01). Postoperative complications such as bile duct injury did not differ between these two groups, however, six (6%) patients in the upfront surgery group died” here. The median time from PC to cholecystectomy was 8 days (range, 0–60 days) (Fig. 1), and no postoperative complications occurred 7 days after PC.

**Table 3:** Characteristics of patients with severe acute cholecystitis with or without preoperative gallbladder drainage

	Upfront surgery (*n* = 100)	PC before cholecystectomy (*n* = 42)	*P*-value
Sex, male/female	59 (59%)/41 (41%)	32 (76%)/10 (24%)	0.05
Age older than 80 years	62 (62%)	23 (55%)	0.42
BMI (kg/m^2^)	24 (13 − 43)	23 (16 − 38)	0.62
ASA-PS ≥ 3	45 (45%)	11 (26%)	**0.04**
CCI	5 (0 − 9)	4 (0 − 9)	0.13
History of biliary infection	7 (7%)	4 (10%)	0.61
WBC (×10^3^/L)	14,435 (2330 − 51,900)	13,560 (1490 − 28,200)	0.94
Hb (g/dL)	12.0 (7.1 − 17.3)	13.0 (8.6 − 17.2)	**0.03**
Alb (g/dL)	2.9 (1.2 − 4.3)	3.1 (2.0 − 4.4)	0.39
CRP (mg/dL)	19.8 (0.5 − 45.5)	20.4 (0.1 − 38.3)	0.48
Warfarin usage	7 (7%)	8 (19%)	**0.03**
DOAC usage	23 (23%)	5 (12%)	0.13
Cardiovascular dysfunction	16 (16%)	14 (33%)	**0.02**
Neurological dysfunction	13 (13%)	7 (17%)	0.57
Respiratory dysfunction	9 (9%)	5 (12%)	0.60
Renal dysfunction	33 (33%)	12 (29%)	0.61
Hepatic dysfunction	37 (37%)	14 (33%)	0.68
Hematological dysfunction	34 (34%)	17 (40%)	0.46
*Preoperative CT findings*			
GB wall thickness	82 (82%)	38 (90%)	0.20
Abdominal abscess	14 (14%)	6 (14%)	0.96
Inflammation around the GB	69 (69%)	34 (81%)	0.17
Enhancement of the GB bed	39 (39%)	16 (38%)	0.64
Duodenal edema	32 (32%)	14 (33%)	0.88
Transverse colon edema	13 (13%)	8 (19%)	0.35
Empyema of the GB	9 (9%)	2 (5%)	0.38
Ascites	35 (35%)	11 (26%)	0.31
Pleural effusion	31 (31%)	12 (29%)	0.75

Abbreviations: Alb, albumin; ASA-PS, American Society of Anesthesiology physical status; BMI, body mass index; CCI, Charlson comorbidity index; CRP, C-reactive protein; CT, computed tomography; DOAC, direct oral anticoagulant; GB, gallbladder; Hb, hemoglobin; WBC, white blood cells; PC, percutaneous cholecystostomy

**Table 4:** Perioperative outcomes of upfront surgery and preoperative GB drainage for patients with severe acute cholecystitis

	Upfront surgery (*n* = 100)	PC before cholecystectomy (*n* = 42)	*P*-value
LC/OC/CC	44/46/10	23/14/5	0.38
Bailout surgery	44 (44%)	17 (40%)	0.20
Intraoperative bleeding (g)	274 (0-3100)	122 (0-500)	**0.04**
Operative time (min)	132 (62–276)	140 (63–314)	0.76
Hospital stay (day)	21 (4–89)	14 (4–45)	**< 0.01**
Blood transfusion	3 (3%)	1 (2%)	**0.84**
Clavien-Dindo classification III-IV	17 (17%)	3 (7%)	0.12
BDI	0	1 (2%)	0.12
90-day mortality	6 (6%)	0 (0%)	0.11
Gangrenous cholecystitis	66 (66%)	21 (50%)	0.07

Abbreviations: BDI, bile duct injury; CC, conversion cholecystectomy; LC, laparoscopic cholecystectomy; OC, open cholecystectomy; PC, percutaneous cholecystostomy

## Discussion

The postoperative complications and hospital stays of patients with grade III AC in this study indicated that both EC and DC were safely performed. Additionally, preoperative PC reduced intraoperative bleeding and the need for blood transfusions; however, upfront surgery did not. Compared to upfront surgery, preoperative PC resulted in significantly shorter postoperative hospital stays. Importantly, patients who underwent preoperative PC did not experience 90-day postoperative mortality. This is the first multi-institutional study to evaluate the optimal surgical timing and efficacy of preoperative PC specifically for grade III AC.

Gutt et al. reported that EC is associated with shorter mean hospital stays and lower total hospital costs compared to those associated with DC [15]. Other studies have observed lower rates of bile duct injury and postoperative complications with EC compared to those observed with DC [4, 5, 16]. Surgical timing reported by several studies ranged from the day of symptom onset to within 7 days after symptom onset; however, those patients had grade I and grade II AC [5, 12, 16, 17]. According to the TG18, antibiotics, hydroelectrolytic disorder correction, and PC are recommended as the initial treatments for grade III AC [6]. EC is strictly limited to advanced laparoscopic centers. Our data suggested that the conversion rates and postoperative complication rates of EC and LC did not differ between patients with grade III AC. EC may be acceptable if the patient’s condition allows for general anesthesia. However, further randomized studies should be performed to evaluate the optimal surgical timing for AC because it is associated with a high mortality rate.

Whether preoperative gallbladder drainage is necessary for patients with grade III AC is uncertain. Compared to LC and OC, PC with tube placement was associated with fewer postoperative complications and lower hospital costs for patients with acalculous AC [18]. Patients who use direct oral anticoagulants and warfarin and require percutaneous transhepatic gallbladder aspiration are at high risk for postprocedural complications such as bleeding. Percutaneous transhepatic gallbladder aspiration is a bridging therapy that allows the safe performance of cholecystectomy. Moreover, percutaneous transhepatic gallbladder aspiration is performed to decompress the gallbladder; therefore, AC recurrence caused by insufficient drainage during percutaneous transhepatic gallbladder aspiration is problematic. However, because percutaneous transhepatic gallbladder aspiration is safe and minimally invasive, it is widely used for patients with AC [19, 20]. Abe et al. reported that preoperative cholecystostomy is useful and safe, especially for grade III AC [21]. Another study reported that patients who underwent PC had higher rates of postprocedural morbidity and mortality and longer hospital stays [22]. This study emphasized that older patients with AC should undergo cholecystectomy unless there are prohibitive surgical risks. Upfront surgery is not optimal for patients who are at high risk for postoperative complications because of their age, ASA physical status, or severe local inflammation. Intraoperative bleeding and blood transfusions were significantly reduced among patients who underwent preoperative PC rather than upfront surgery. Preoperative PC can ease severe local inflammation caused by gallbladder drainage and strongly reduce intraoperative bleeding.

Bile duct injury is the most alarming complication. When bile duct injury occurs, repeated ERCP, liver resection, and liver transplantation could be required. BOS comprises various procedures, including conversion from LC to open surgery, the fundus-first approach, and subtotal cholecystectomy. However, the optimal approach is unclear. Conrad et al. recommended the fundus-first approach with partial cholecystectomy to avoid bile duct injury [23]. Compared with the ordinal approach, the fundus-first approach can reduce the incidence of bile duct injury [24]. In contrast, subtotal cholecystectomy is the most efficacious and safest procedure for preventing bile duct injury when a CVS cannot be achieved. During our previous study, we found that laparoscopic BOS, including that comprising the fundus-first approach or subtotal approach, was associated with less excessive intraoperative bleeding and shorter hospital stays compared to those associated with conversion surgery. During this study, approximately 50% of cases required BOS, and this may have contributed to the prevention of bile duct injury associated with grade III AC.

Although this study had some strengths, such as the use of a multi-institutional database, it also had some limitations. This was a retrospective study with a relatively small number of patients. The attending surgeons chose the surgical approach and determined whether to perform gallbladder drainage or upfront surgery based on their previous experience. Surgical timing after gallbladder drainage was strongly dependent on the policy of the facility. Further prospective studies should be performed to evaluate the optimal surgical timing and efficacy of preoperative gallbladder drainage for grade III AC.

In conclusion, preoperative PC can prevent excessive bleeding and shorten the postoperative hospital stay. Performing LC after PC during the same hospitalization can help prevent AC recurrence. When a surgical approach to necrotizing cholecystitis is required and conservative treatment is unsuccessful, EC should be considered.

## Data Availability

The datasets used and/or during current study are available from the corresponding author on reasonable request.
